# The association of speckle-type POZ protein with lymph node metastasis and prognosis in cancer patients: A meta-analysis

**DOI:** 10.1097/MD.0000000000017439

**Published:** 2019-10-04

**Authors:** Fei Cheng, Chunyan Zeng, Ling Zeng, Chayan Wu, Youxiang Chen

**Affiliations:** Departments of Gastroenterology, The First Affiliated Hospital of Nanchang University, Nanchang, Jiangxi, China.

**Keywords:** cancer, meta-analysis, prognosis, SPOP

## Abstract

**Background:**

Speckle-type POZ protein (SPOP) has recently been reported as a prognostic tumor biomarker. However, the predictive value of SPOP remains controversial in human cancers. The current meta-analysis was performed to obtain a comprehensive evaluation of the relationship between SPOP expression and prognosis of cancer patients.

**Methods:**

Embase, Pubmed, Web of Science, and Chinese Biomedical Literature database were systematically searched up to January 2, 2019. The pooled hazard ratios (HRs) and/or pooled odds ratios (ORs) with 95% confidence intervals (CIs) were used to quantitatively assess the relationship of SPOP expression with prognosis and lymph node metastasis (LNM).

**Results:**

A total of 9 studies with 928 patients were included in this meta-analysis. The results showed that low SPOP expression was significantly related to poor overall survival (high/low: HR = 0.55; 95% CI: 0.38–0.79, *P* = .001), especially for digestive system cancers (high/low: HR = 0.46; 95% CI: 0.27–0.78, *P* = .003). However, SPOP expression did not affect progression-free survival in cancer patients (high/low: HR = 2.07; 95% CI: 0.16–26.70, *P* = .578). Additionally, the association between SPOP overexpression and LNM was positive in patients with clear cell renal cell carcinoma (ccRCC) (OR = 5.26; 95% CI: 1.66–16.68, *P* = .005) but negative in cancer patients without ccRCC (OR = 0.36; 95% CI: 0.21–0.62, *P* < .001).

**Conclusion:**

Decreased SPOP expression could predict poor prognosis of cancer patients, suggesting that SPOP protein may be a useful prognostic biomarker in cancer patients.

## Abstract 2

Supplemental Digital Content is available in the text

## 1 Introduction

Cancer is one of the leading causes of death worldwide and has become a major public health event. In 2018, there were nearly 2 million new cancer cases and 609 thousand cancer-related deaths in the United States.^[1]^ Although the overall survival (OS) rates for patients with malignant neoplasm have improved due to the widespread implementation of early cancer screening and radical surgery, numerous patients with cancers are still diagnosed at advanced stages, and subsequently, it is difficult to reverse the poor outcome. Hence, it is imperative to develop novel biomarkers to predict cancer prognosis.

Ubiquitin-dependent proteolysis system plays a crucial role in the regulation of a variety of cellular processes, including cell proliferation and apoptosis.^[2]^ In this system, the addition of ubiquitin to target protein is mediated by a cascade of enzymatic reactions consisting of an E1 activating enzyme, an E2 conjugation enzyme, and an E3 ubiquitin ligase, of which substrate specificity depends on E3 ubiquitin ligase.^[3]^ Furthermore, the complex interactions between E1, E2, and E3 ubiquitin ligase bring various substrates to be modified and in turn contribute to the ubiquitin-mediated degradation of substrates. Perhaps Cullin-RING E3 ubiquitin ligase (CRL3) including a molecular scaffold (Cullin) is the most important among the E3 ligase family. Recent evidence strongly suggested that CRL3 exerts pivotal roles in the regulation of disease conditions, including cancer progression.^[4]^ Moreover, CRL3 recruits specific substrates through the binding of Cullins to their substrate-binding adaptors.

Speckle-type POZ protein (SPOP) is a unique adaptor of CRL3 that includes an N-terminal MATH domain, a BTB/POZ domain, and a C-terminal nuclear localization sequence.^[5]^ It is well known that SPOP functions as a major executor of the proteasome-mediated degradation of certain substrates. Recently, mounting evidence suggested that downregulation of SPOP expression extensively occurs in various tumor tissues due to mutations and DNA methylations.^[6,7]^ Ju et al demonstrated that SPOP exhibits a tumor repressor role via targeting cyclin E1 and promotes the cell proliferation, migration, and tumor formation.^[8]^ Similarly, Groner et al reported that SPOP, but not its mutants, promotes the ubiquitination and degradation of TRIM24 and hereby inhibits tumorigenesis and development of tumor.^[9]^ Furthermore, SPOP has also been found to have potential prognostic value in a variety of tumors. Multiple studies confirmed a significant correlation between decreased SPOP expression and poor prognosis in cancer patients,^[10–15]^ while the opposite results were observed in other studies.^[16–18]^ Since the prognostic relevance of SPOP expression in cancers remains controversial, it is essential to perform a meta-analysis to systematically evaluate the prognostic role of SPOP in cancer patients.

## 2 Materials and methods

### 2.1 Search strategy

Following the preferred reporting items for systematic reviews and meta-analyses statement, Embase, Pubmed, Web of Science, and Chinese Biomedical Literature database were searched to retrieve potential studies on the theme up to January 2, 2019. The articles were identified using the following search strategy: (“speckle-type POZ protein” or “SPOP”) and (“survival” or “prognosis”) and (“cancer” or “tumor” or “neoplasm” or “carcinoma”). The published languages were restricted to English and Chinese. Additionally, citation lists of eligible articles were also searched manually for possible inclusion. Two investigators conducted literature collection independently. Ethical approval and patient consent were not required for this meta-analysis.

### 2.2 Study selection criteria

Studies were eligible for inclusion based on the following criteria: identified studies focused on the association between SPOP expression and prognosis and lymph node metastasis (LNM) in human cancer; the hazard ratio (HR) and its 95% confidence interval (CI) could be extracted for OS or progression-free survival (PFS); studies were published in English or Chinese; and patients were divided into high and low expression groups according to the cut-off value of SPOP. The exclusion criteria were as follows: insufficient data available to assess outcomes; studies with overlapping data; and reviews, letters, conference abstracts, and case reports.

### 2.3 Data extraction and quality assessment

The following information was independently extracted by 2 investigators: first author, publication year, study country, cancer type, sample size, detection method, sample source, LNM, outcome, follow-up period, cut-off value, antibody, and HR with 95% CI. If HRs and 95% CI were not directly reported, they were recalculated by the data extracted from Kaplan–Meier survival curves (SC) using Engauge Digitizer version V4.1.^[19]^ We selected multivariate result if univariate and multivariate results were both provided. Additionally, we evaluated study quality following the Newcastle–Ottawa scale (NOS) including 3 main categories with 8 items, which scores ranged from 0 to 9. A study with an NOS score ≥6 was considered high quality; otherwise, studies were regarded as low-quality studies.

### 2.4 Statistical analysis

The meta-analyses were performed by Stata 12.0 software and Review Manager 5.3. The effect of SPOP expression on prognosis (OS and PFS) of cancer was calculated as pooled HRs with 95% CI. An observed HR (high/low) <1 indicated a poor prognosis for patients with low SPOP expression. Odds ratios (ORs) and their 95% CIs were combined to evaluate the association between SPOP expression and LNM. Statistical heterogeneity was assessed using the *I*^2^ index and Chi-square test (assessing the *P*-value). If *P* > .05 or *I*^2^ < 50%, indicating no significant heterogeneity, the fixed-effects model was chosen. Otherwise, we used the random-effects model. Subgroup analyses and the Galbraith plots were performed to further explore the potential source of heterogeneity. Begg test and Egger test were performed to evaluate the publication bias. Statistical significance was defined as *P* < .05.

## 3 Results

### 3.1 Literature information and study characteristics

**Figure 1:**
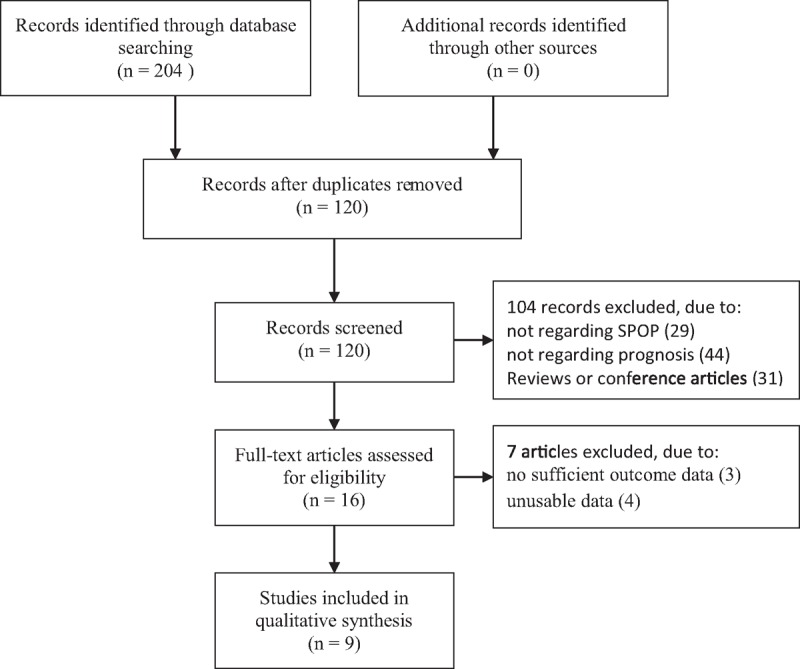
Flowchart presenting the steps in the literature search and selection.

Approximately 204 publications, of which 84 were duplicate studies, were found from the database search by using the search strategy above (Fig. 1). One hundred four articles were directly excluded by screening the titles and abstracts. Next, a total of 16 studies were assessed for eligibility by reading the full-text, 7 of which were excluded for insufficient outcome data and unusable data. Eventually, 9 studies were enrolled in the present meta-analysis, including 928 cases.^[10–18]^ The main information obtained from the included studies was shown in Table 1. All studies included in the present meta-analysis were retrospective studies published between 2014 and 2018. The sample sizes ranged from 44 to 265 cases, and the follow-up period ranged from 60 to 189 months. Seven different types of cancer were evaluated in the meta-analysis, with 3 clear cell renal cell carcinoma (ccRCC), 1 non-small cell lung cancer (NSCLC), 1 glioma, 1 gastric cancer, 1 prostate cancer, 1 colorectal cancer (CRC), and 1 hepatocellular carcinoma (HCC). Of these studies, 7 originated from China, 1 from Spain, and 1 from Egypt. Seven studies used the immunohistochemistry (IHC) staining method, and 2 studies applied reverse transcription-polymerase chain reaction (RT-PCR). HRs based on univariate and multivariate method for OS were reported in 4 studies and 6 studies, respectively. HRs and 95% CIs for OS were provided directly in 6 studies and extracted from Kaplan–Meier SC in the other study. Five studies presented the correlation between SPOP expression and LNM. Following the NOS, all including studies were high-quality studies with score ≥6.

**Table 1:**
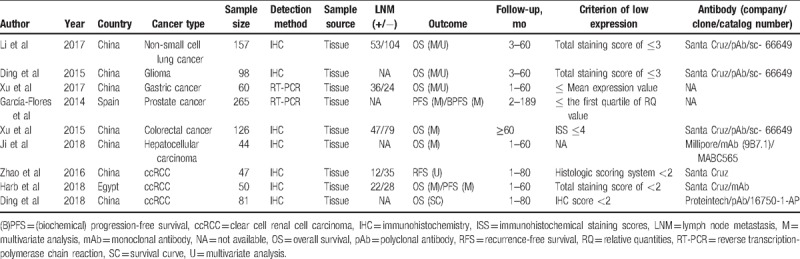
Main characteristics of included studies in the meta-analysis.

### 3.2 The prognostic value of SPOP in OS

**Figure 2:**
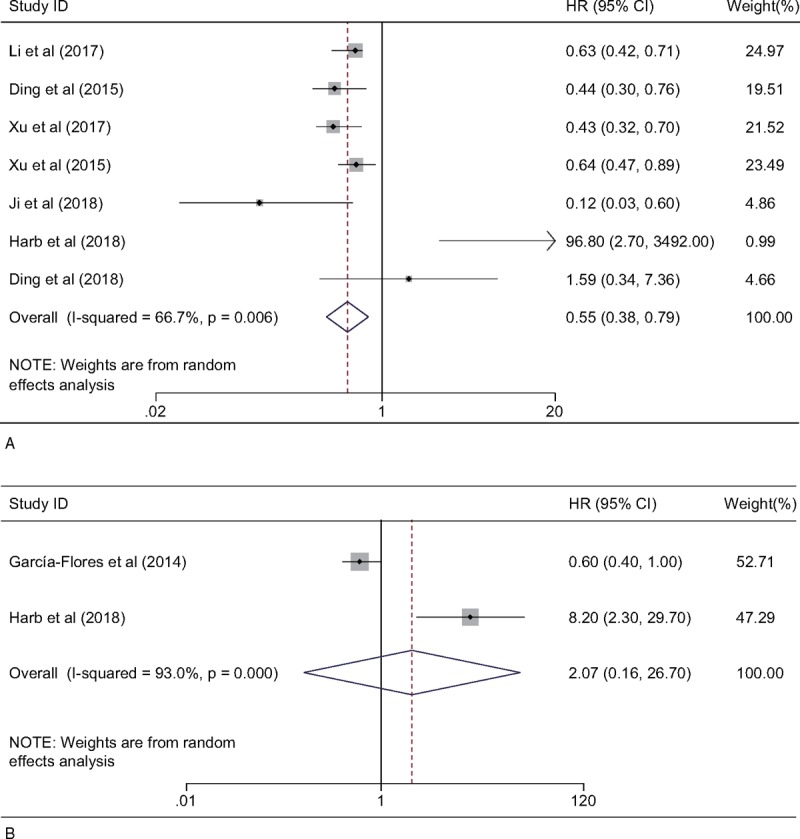
Forest plot showing the pooled HR for the relationship between SPOP expression and OS (A) or PFS (B) in cancer patients. HR = hazard ratio, OS = overall survival, PFS = progression-free survival, SPOP = speckle-type POZ protein.

A total of 7 studies were enrolled to explore whether SPOP was an independent predictive factor for OS in cancer patients. Considering the heterogeneity among studies in evaluating OS (*I*^2^ = 66.7%, *P* = .006), a random-effects model was applied to pool the HRs. The pooled HRs revealed that low expression of SPOP was significantly related to a shorter OS (high/low: HR = 0.55; 95% CI: 0.38–0.79, *P* = .001) (Fig. 2A). To further explore the sources of high heterogeneity, subgroup analyses were performed by cancer type, sample size, publication year, and country (Table 2). The subgroup analysis according to cancer type demonstrated that low expression of SPOP was related to poor OS in digestive system cancers (high/low: HR = 0.46; 95% CI: 0.27–0.78, *P* = .003). Moreover, subgroup analysis based on the sample size indicated that low SPOP expression was a poor prognostic biomarker in the large sample size group (n ≥ 90, high/low: HR = 0.60; 95% CI: 0.50–0.72, *P* < .001). In a subgroup analysis according to publication year, similar pooled HRs were also observed in the studies published between 2015 and 2017 (high/low: HR = 0.56; 95% CI: 0.48–0.67, *P* < .001). Finally, the country subgroup analysis verified the positive impact of decreased SPOP expression on adverse OS in Asian countries (high/low: HR = 0.53; 95% CI: 0.41–0.70, *P* < .001). No significant differences were observed in any other subgroup analysis. As shown in the Galbraith plots (Supplemental Fig. 1), the studies published by Harb et al^[16]^ and Ji et al^[12]^ should be the main contributors to heterogeneity in OS.

**Table 2:**
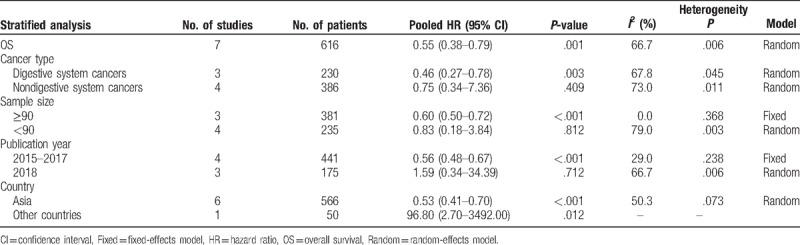
Subgroup analysis of pooled HRs for OS in cancer patients with high SPOP expression.

### 3.3 The prognostic value of SPOP in PFS

Only 2 studies included a total of 315 cases from Spain and Egypt, providing suitable information for PFS analyses. Since the studies evaluating PFS with aberrant SPOP expression were of severe heterogeneity (*I*^2^ = 93.0%; *P* < .001), a random-effects model was used to pool the HRs, and no statistically significant relevance was observed (high/low: HR = 2.07; 95% CI: 0.16–26.70, *P* = .578) (Fig. 2B).

### 3.4 Correlation between SPOP and LNM

**Figure 3:**
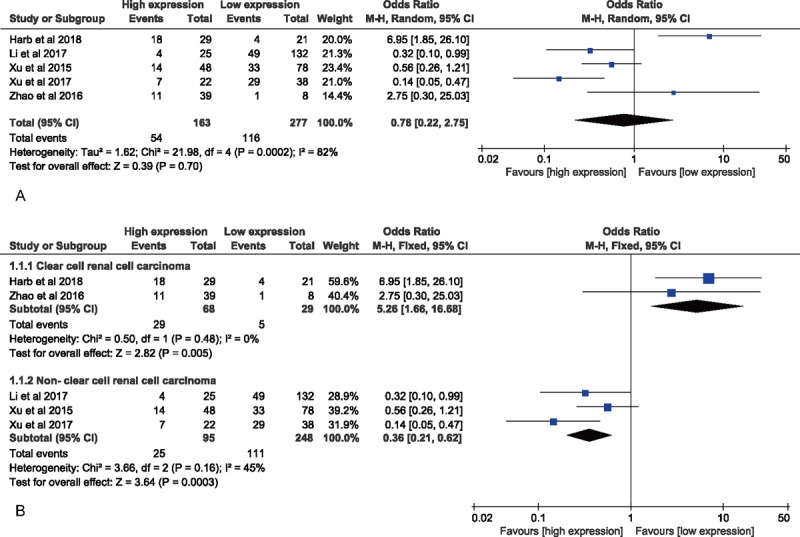
Forest plot for evaluating the relationship between SPOP expression and LNM. (A) Forest plot to assess the overall effect; (B) Forest plots for the subgroup analysis based on cancer type. LNM = lymph node metastasis, SPOP = speckle-type POZ protein.

For studies evaluating LNM for SPOP in the 5 cohorts, a random-effects model was used to calculate the pooled OR and its 95% CI due to the highly significant heterogeneity (*I*^2^ = 82.0%; *P* < .001) (Fig. 3A). The result might predict that elevated SPOP expression was negatively associated with LNM based on the pooled OR (OR = 0.78; 95% CI: 0.22–2.75), but the effect did not reach the level of statistical significance (*P* = .700). However, further stratified analyses according to cancer type demonstrated the relationship between SPOP overexpression and LNM was positive in ccRCC (OR = 5.26; 95% CI: 1.66–16.68, *P* = .005, fixed-effect model) but negative in non-ccRCC (OR = 0.36; 95% CI: 0.21–0.62, *P* < .001, fixed-effect model) (Fig. 3B).

### 3.5 Sensitivity analysis and publication bias

**Figure 4:**
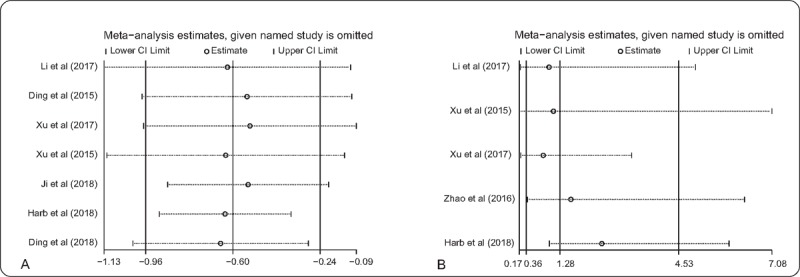
Sensitivity analysis. (A) Effect of individual studies on the pooled HR for OS associated with SPOP expression; (B) effect of individual studies on the pooled OR for LNM associated with SPOP expression. HR = hazard ratio, LNM = lymph node metastasis, OR = odds ratios, OS = overall survival, SPOP = speckle-type POZ protein.

**Figure 5:**
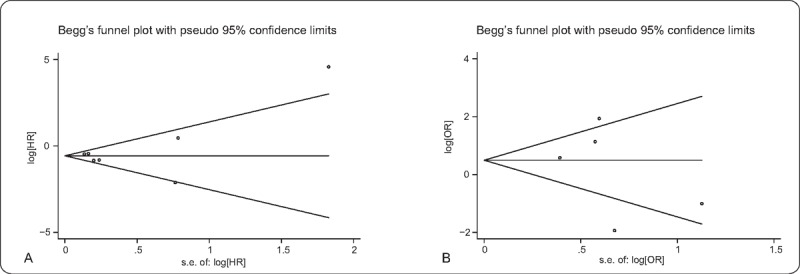
Begg's funnel plot. (A) Effect of SPOP expression on OS; (B) effect of SPOP expression on LNM. LNM = lymph node metastasis, OS = overall survival, SPOP = speckle-type POZ protein.

To assess the influence of an individual study on the robustness of overall result, sensitivity analyses were conducted by sequentially omitting each study. In sensitivity analyses of SPOP expression involved in OS (Fig. 4A) and LNM (Fig. 4B), neither pooled results showed obvious variation, and thus confirmed the stability of the studies. In addition, our results indicated that there was no evidence of significant publication bias in 7 cohorts evaluating OS (Begg *P* = .764 and Egger *P* = .667) (Fig. 5A) and in 5 cohorts evaluating LNM (Begg *P* = .806 and Egger *P* = .532) (Fig. 5B).

## 4 Discussion

Recently, whole-exome sequencing studies revealed that genomic mutations in SPOP are frequently present in various tumors, and interestingly, in prostate and endometrial cancer, all mutation regions of SPOP are localized to its MATH domain.^[20,21]^ In addition to mutations, epigenetic silencing also widely contributes to the downregulation of SPOP expression. Zhi et al reported that the core region of SPOP promoter was hypermethylated in CRC, which affected the binding affinity between transcription factor RXRA and SPOP promoter, culminating in overexpression of oncogenic target, Gli2.^[22]^ Similarly, in NSCLC, hypermethylation of CpG islands of SPOP prevented it from binding to another transcription factor, C/EBPα, and promoted invasion, migration, proliferation in vitro and tumor growth in vivo.^[6]^ SPOP, as a vital suppressor of oncogene, has drawn wide attentions for implicating a potential prognostic value and therapeutic target for future medication. It was reported that decreased expression of SPOP consistently occurred in glioma patients, which was positively correlated with advanced tumor grade and worse survival.^[14]^ The same prognostic result was observed in NSCLC, and the downregulation of SPOP expression contributed to poor tumor differentiation and LNM.^[11]^ Additionally, the expression level of SPOP, identified as a prognosis-related biomarker, was significantly lower in CRC tissues than adjacent normal tissues.^[13]^ Although the majority of studies focused on the potential evidence indicating an unfavorable impact of low SPOP expression on clinical outcome, the prognostic value of SPOP in cancer patients is still controversial. In contrast, several studies recently highlighted that a favorable survival duration was obtained in kidney cancer patients with low SPOP expression, although the mechanism was not fully elucidated.^[16,17]^ Thus, a comprehensive study is urgently needed.

To date, our meta-analysis is the first to investigate the relationship between low SPOP expression and clinical prognostic value in 9 studies with 928 cancer patients. Our results showed that low SPOP expression was correlated with worse survival in patients with various carcinomas, indicating that SPOP may act as a potential prognostic marker. In the subgroup analyses, the adverse prognostic role of decreased SPOP expression remained significant in digestive system cancers, the large sample size group, studies performed in Asian countries and those published between 2015 and 2017. To further identify potential sources of heterogeneity, a Galbraith plot was created and showed that marked heterogeneity was mainly attributed to those 2 studies with small sample size, which were both published in 2018 by Harb et al^[16]^ and Ji et al.^[12]^ Additionally, the existing evidence in our meta-analysis was insufficient to verify the definitive association between SPOP and PFS. Our findings might imply that SPOP is not a significant prognostic indicator for PFS in cancer patients. Nevertheless, this result should be interpreted with caution due to the limited data and severe heterogeneity among the included studies. Therefore, future studies with large sample sizes are required to synthetically evaluate the prognostic value of SPOP in PFS.

The underlying mechanisms involved in the relationship between low SPOP expression and poor prognosis of cancer patients have been universally investigated. Further supporting the evidence of SPOP as a tumor repressor is the steadily increasing number of SPOP substrates shown in Table 3, most of which are known oncogenes. Particularly in prostate cancer, previous publications had reported an abundance of substrate proteins because of recurrent SPOP mutations. Androgen receptor (AR), a member of the nuclear receptor superfamily, is crucial for normal prostate cell growth and survival. In 2014, An et al first discovered that wild-type SPOP was a direct regulator of AR via ubiquitination.^[23]^ steroid receptor coactivator-3, a preferred co-activator for hormone-activated AR, is another classic SPOP substrate. Geng et al reported that SPOP mutants lost the ability to modulate SRC-3 and AR transcriptional activity.^[24]^ A follow-up study also showed that SPOP mutation drove prostate tumorigenesis depending on coactivation of both SRC-3-mediated phosphatidylinositol-3 -kinase (PI3K)/mammalian target of rapamycin (mTOR) and AR signaling.^[25]^ A study in 2018 by Zhang et al demonstrated that SPOP promotes the ubiquitin-mediated degradation of programmed death-ligand 1, a promising target for immunotherapy. They also highlighted that mutant SPOP leads to elevated programmed death-ligand 1 levels and reduced numbers of CD8+ tumor-infiltrating lymphocytes in prostate cancer.^[26]^ In breast cancer, a loss of heterozygosity of SPOP frequently contributes to breast cancer cell growth and invasive phenotype via targeting progesterone receptor.^[27]^ The ectopic expression of SPOP also affects primary tumorigenesis by targeting epithelial-mesenchymal transition (EMT)-inducing C-Myc.^[28]^ In HCC, Ji et al verified that increased degradation of SUMO1/sentrin specific peptidase 7 by restoration of SPOP decreased vimentin levels, which in turn attenuated HCC cell metastasis.^[12]^ In gastric cancer, SPOP was consistently downregulated and inhibited gastric cancer cell proliferation and migration via targeting Gli2 in the hedgehog pathway.^[29]^ A recent study also found the antitumor effect of SPOP in colon cancer cell line HCT116 by targeting HDAC6.^[30]^ Additionally, Luo et al in 2017 showed that SPOP mutation protected SIRT2 protein from ubiquitination in NSCLC.^[31]^ In 2018, the author also found that FAS-associated protein with death domain was directly downregulated by SPOP and thus inactivate apoptosis-related nuclear factor kappa B signaling.^[32]^ However, SPOP does not always represent a tumor suppressor. In ccRCC, Zhao et al verified that SPOP functions as an activator of β-catenin/T-cell factor 4 (TCF4) signaling and promoted cell invasion and EMT.^[17]^ Similarly, Li et al discovered that accumulating SPOP expression induced by hypoxia promotes tumorigenesis by regulating the degradation of Daxx, phosphatase and tensin homolog deleted on chromosome 10, and dual specificity phosphatase 7.^[33]^ Based on these findings, the exact mechanisms for the anti- or pro-tumor effects of SPOP in different tumors deserve further confirmation.

**Table 3:**
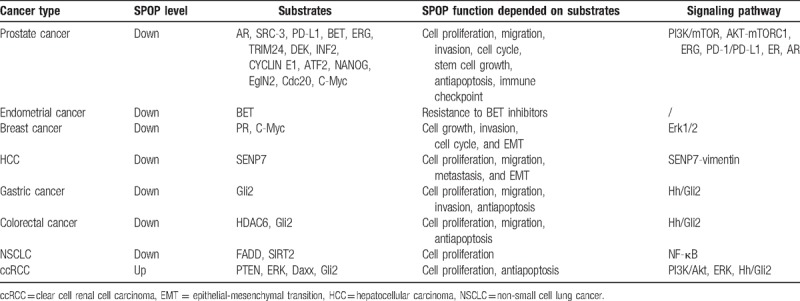
The dysregulated expression and validated substrates of SPOP in cancers.

We also assessed the underlying impact of SPOP on LNM, which possessed a crucial role in tumor recurrence and survival of cancer patients. For this meta-analysis, the overall pooled result including 5 eligible studies did not predict a significant relationship between SPOP expression and LNM in cancers. Interestingly, by further stratified analyses according to cancer type, our findings indicated that SPOP overexpression exhibited a close correlation with LNM in ccRCC. Conversely, significant results also revealed that low expression of SPOP was positively associated with LNM in non-ccRCC, such as CRC, gastric cancer, and NSCLC. Presumably, the selection of specific pathological types of cancer patients might lead to the result of ccRCC patients opposite to those of non-ccRCC patients. For example, the results from Zhao et al^[17]^ and Harb et al^[16]^ demonstrated the positive association between elevated SPOP expression and LNM that occurred only in patients with ccRCC, not including patients with all subtypes of renal cell carcinoma (RCC). More importantly, Zhao et al^[17]^ verified that SPOP is highly expressed in ccRCC but not or weakly expressed in other subtypes of RCC, such as papillary, chromophobe or oncocytoma RCC, indicating a discrepancy among SPOP expression in different types of the same tumor. However, 2 studies by the same author, Xu et al,^[10,13]^ showed that the analyses of eligible studies were not performed only according to certain specific tumor subtypes in non-ccRCC group.

Admittedly, there are some limitations for this meta-analysis. First, given that the biological functions of SPOP in cancers could vary, pooled analysis in single cancer type might be useful to specifically evaluate the prognostic role of SPOP in a certain type of tumor. However, the subgroup analysis based on single cancer type was not conducted due to limitation of the number of cohorts included. Besides, there were only 2 studies on PFS. Thus, more studies are warranted to exactly determine the prognostic value of SPOP expression in various malignancies. Second, the inconsistent cut-off values of SPOP expression might impact on the precision of the prognostic role of SPOP in human cancer. Third, although heterogeneity was modified by the application of a random-effects model and subgroup analysis, there was still some heterogeneity among some subgroups. Fourth, both studies from same region were published by the same author, Xu et al,^[10,13]^ bringing a few selective deviations despite enrolling patients with different tumor types in various cohorts. Fifth, 2 studies examined SPOP expression by RT-PCR, which differed from the IHC method widely used in other studies. Finally, since some HRs were determined by the data extracted from SC, a tiny discrepancy might exist between the actual HRs and the estimated data.

In conclusion, this meta-analysis clarified that decreased SPOP expression predicts poor OS in cancer patients, and positively correlates with LNM in cancer patients without ccRCC rather than in cancer patients with ccRCC. It might be suggested that SPOP can serve as a promising biomarker for predicting the prognosis of cancer patients. Considering that several limitations existed, it should be cautious to appreciate the conclusion, and well-designed, large-scale studies are imperative to verify the biological and prognostic significance of SPOP in cancers, especially in a single type of cancer.

## Author contributions

**Conceptualization:** Fei Cheng, Youxiang Chen.

**Data curation:** Fei Cheng, Ling Zeng, Chayan Wu.

**Formal analysis:** Fei Cheng, Chunyan Zeng.

**Funding acquisition:** Chunyan Zeng, Youxiang Chen.

**Methodology:** Youxiang Chen.

**Software:** Fei Cheng.

**Supervision:** Youxiang Chen.

**Writing – original draft:** Fei Cheng, Chunyan Zeng.

**Writing – review & editing:** Youxiang Chen.

## Supplementary Material

Supplemental Digital Content
